# Population Preferences for Treatment in Life-Limiting Illness: Valuing the Way Time Is Spent at the End of Life

**DOI:** 10.1177/0272989X251346203

**Published:** 2025-07-10

**Authors:** Patricia Kenny, Deborah J. Street, Jane Hall

**Affiliations:** University of Technology Sydney (Centre for Health Economics Research & Evaluation [CHERE]), Sydney, Australia; University of Technology Sydney (Centre for Health Economics Research & Evaluation [CHERE]), Sydney, Australia; University of Technology Sydney (Centre for Health Economics Research & Evaluation [CHERE]), Sydney, Australia

**Keywords:** Discrete choice experiment, palliative care, community preferences

## Abstract

**Introduction:**

Societal preferences over different health states are used to guide service planning, but there has been little investigation of treatment preferences at the end of life. This study aimed to examine population preferences for active treatment or palliation for cancer patients when life expectancy is limited and the relative importance of time spent in hospital or with functional limitation.

**Methods:**

We used a discrete choice experiment that presented respondents with a series of hypothetical patients who had died, describing their last few months of life. Respondents selected the end-of-life alternative they thought best. Data were collected from 1,502 Australian adults participating in an online survey panel. Latent class analysis was used to identify groups with different preference patterns.

**Results:**

Four preference groups were identified along with an additional group that we termed *inattentive*, as they appeared to respond at random. Among the 1,070 respondents assigned to 1 of the 4 preference groups, 33.5% favored longer overall survival regardless of how that time was spent; 26.1% were willing to accept a shorter survival time for less time in the hospital or completely incapacitated at home, and they had a stronger preference for palliative care in older patients; 22.5% strongly supported the use of palliative care regardless of the age of the patients, preferring less time in the hospital or time at home with any functional limitations; and 17.9% had a strong preference to not use palliative care.

**Conclusions:**

Our results show distinct heterogeneity in population preferences for end-of-life care. Policy goals and service planning should acknowledge this heterogeneity and provide end-of-life support services that offer the flexibility to enhance patient choice. Many current funding approaches are not consistent with the philosophy of patient-centered care. Policy makers can and should be exploring innovative approaches to improve efficiency and equity.

**Highlights:**

- Social preferences, based on a general population survey, vary across palliative and active care approaches.
- Preferences for palliative care and willingness to tolerate time in hospital and time at home with activity limitations varied within the groups willing to trade quality and quantity of life.
- Policy, resource allocation, and funding methods should accommodate this variability.

The costs of health care generally increase toward the end of life,^1–7^ with some studies attributing a part of this to the use of futile or nonbeneficial treatments at the end of life.^2,8,9^ The use of such treatments may also lead to more discomfort and poorer quality of life for people with a life-limiting illness. On the other hand, some patients and their families may wish to pursue every available treatment even with a high risk of toxic effects, suggesting a high value of hope.^10^ The use of chemotherapy in the final weeks or months of life has been identified as one measure of futile or nonbeneficial treatment,^11^ although there is considerable variation in defining what is nonbeneficial or futile care.^11,12^

Patient-centered care is “providing care that is respectful of and responsive to individual patient preferences, needs and values and ensuring patient values guide all clinical decisions” (Institute of Medicine).^13^ It has increasingly been endorsed as an attribute of health care quality (World Health Organization).^14^ For the most part, this has been focused on the clinical level, that is, the interaction between the patient and the clinical team, ensuring that patient needs and wishes are respected. However, patient or person centeredness should also affect decision making at the level of organizations (meso level) and governance and funding (macro).^15^

We are interested in policy and planning of services for end-of-life care and how this can improve patient care and well-being. Standardization of care can come through funding, where some services are reimbursed while others are not, and is not responsive to individual preferences, particularly when these differ from average.^15^ The financial incentives imposed by these funding decisions can lead to suboptimal care, for example, encouraging hospital admissions over care at home.^16^ If policy and planning are to incorporate patient centeredness at the meso and macro levels, it is important to understand and measure preferences and explore heterogeneity.

Treatments for advanced cancer sometimes offer the possibility of extending survival by a relatively small amount.^17,18^ The typical trajectory for people at the end of life will involve fluctuations in health state and may involve substantial amounts of time attending hospital for treatment or being admitted to hospital. They also may be considerably disabled when at home due to treatment side effects or disease. This treatment-related time burden has been described as the “time toxicity” of cancer treatment.^19^

In this study, the extent to which people are willing to trade quantity and quality of life is explored. Further understanding is provided by identifying the value attached to the relative balance between additional survival time and the time toxicity and levels of disability directly associated with treatment. We test whether societal values differ for treatment according to the age of patients, hypothesizing that people would favor more active treatment for younger patients. We also explore heterogeneity in preferences, hypothesizing that there will be a group for whom extension of life is dominant and who reject palliative care. We recruit a community sample of respondents, to reflect societal values, consistent with most other societal preference elicitation approaches.

A discrete choice experiment (DCE) is an approach to eliciting preferences that allows respondents to make tradeoffs between characteristics and outcomes of treatments. Unsurprisingly, quality of life and length of life are among the attributes that have been identified as important attributes in several DCE studies of preferences for care at the end of life among patient and population samples.^20^ Studies among small samples of people with advanced cancer found that willingness to pay to avoid severe pain was similar to that for a 1-y life extension^21^ and that willingness to pay to improve quality of life from poor to very good was higher than for a life extension of 10 mo.^22^ Finkelstein et al.^23^ identified 3 groups with different preferences among adults with cancer: a group favoring palliative care, a group favoring life-extending treatment, and a group willing to trade quantity and quality of life. Reed et al.^24^ identified 4 preference groups among cancer patients with groups differing in terms of preferences for treatments offering different levels of short- and long-term survival, costs, and physical functioning. We found no DCE studies that explicitly considered how people valued time spent in hospital or at home with different levels of functioning, which may affect the value members of society attach to treatments.

We used a DCE to understand Australian population preferences in relation to the importance of extended survival over quality of life and the value attached to the way time is spent in the context of limited life expectancy. We identify population preferences as derived from community samples, rather than patients and/or their carers. The study aimed to examine preferences for active or palliative management of advanced cancer over the last year of life and the relative importance of time spent in hospital or at home, as well as time spent at home with different levels of disability. Instead of asking individuals which treatment they would choose for themselves, we assessed population preferences based on hypothetical patients with advanced cancer who had died after different treatments (which we describe as different trajectories) and also considered how preferences varied when the hypothetical patient was at different ages and life stages.

## Methods

We conducted a DCE to assess Australian population preferences for palliative care or chemotherapy for the management of advanced cancer, where chemotherapy offered a range of different survival and other outcomes. The DCE asked participants to choose between different completed trajectories with different levels of survival, time in hospital. and periods with disability when not in hospital. It also included 3 different patient vignettes with patients at different ages and life stages, as we hypothesized that societal preferences would assign a higher value to active treatment for younger patients but to prefer palliative care for older patients who could be considered to have had a “fair innings.”^25^

### Sample

The sample included 1,502 Australian adults aged 18 y or older from the Australian general population, recruited through the Cint online panel during September to October 2023. Recruitment quotas were used to ensure the sample represented Australian adults in terms of age and gender. The survey was built and implemented by SurveyEngine, and despite the use of measures to detect bots or disengaged respondents, 48 of the initial respondents were replaced with additional sampling due to open-ended text responses that indicated nongenuine responses. We had decided on a general population sample rather than a patient or carer sample to represent the range of values and attitudes in the community, similar to other preference studies that use community values rather than those of patients to guide resource allocation. Furthermore, there is evidence that patient preferences for the place of care and death change as disease progresses,^26–28^ suggesting shifting priorities as needs change. This makes single point-in-time patient preferences a less useful basis for health care policy (although essential for individual decision making at that time). Consequently, both policy setting and program evaluation should recognize multiple goals over the course of the end-of-life period. A community sample valuing the end-of-life trajectory (including time spent in different states) may differently and usefully inform overall policy relative to a patient sample strongly influenced by their current place in that trajectory.

### Survey

The survey included a range of sociodemographic and attitudinal questions, in addition to the DCE section. There was also explanatory material covering the purpose of the study, information about different kinds of care toward the end of life, as well as a description of the DCE questions and how to answer them (including an example). There was also an explanation about how to access additional explanatory information using popups. The attitudinal questions asked about opinions on some different approaches to the care of someone with a life-limiting illness such as opinions about voluntary assisted dying or preferred goals of care. The demographic and attitudinal questions were included to describe the sample and for examining the impact of these variables on preferences.

### DCE Section

The DCE section of the survey focused on survival time and the way the remaining survival time was spent under 3 hypothetical treatment regimes: 1 palliative care and 2 chemotherapy treatments. This section of the survey included 15 choice questions, each asking which of 3 completed treatment trajectories the respondent thought was best and which was worst. (We use only the best responses.) We presented completed trajectories with known outcomes so as to remove the interpretation of probabilities (in survival and side effects) and to facilitate the evaluation of the overall relative time spent in hospital or at home in different health states as that can be known only retrospectively. There were 5 attributes, 2 related to time spent at the hospital and 3 related to time spent at home, where home time describes time not admitted to, or attending, the hospital. The 2 hospital attributes represented time spent as an inpatient (for management of treatment side effects or disease symptoms) and time spent attending the hospital (for treatment or tests). The 3 home-related attributes represented time spent with different levels of disability due to disease or treatment; these were able to do all usual activities, able to do some usual activities, or not able to do any activities. The levels of the attributes comprise different time periods (in months) spent in each state.

**Figure 1:**
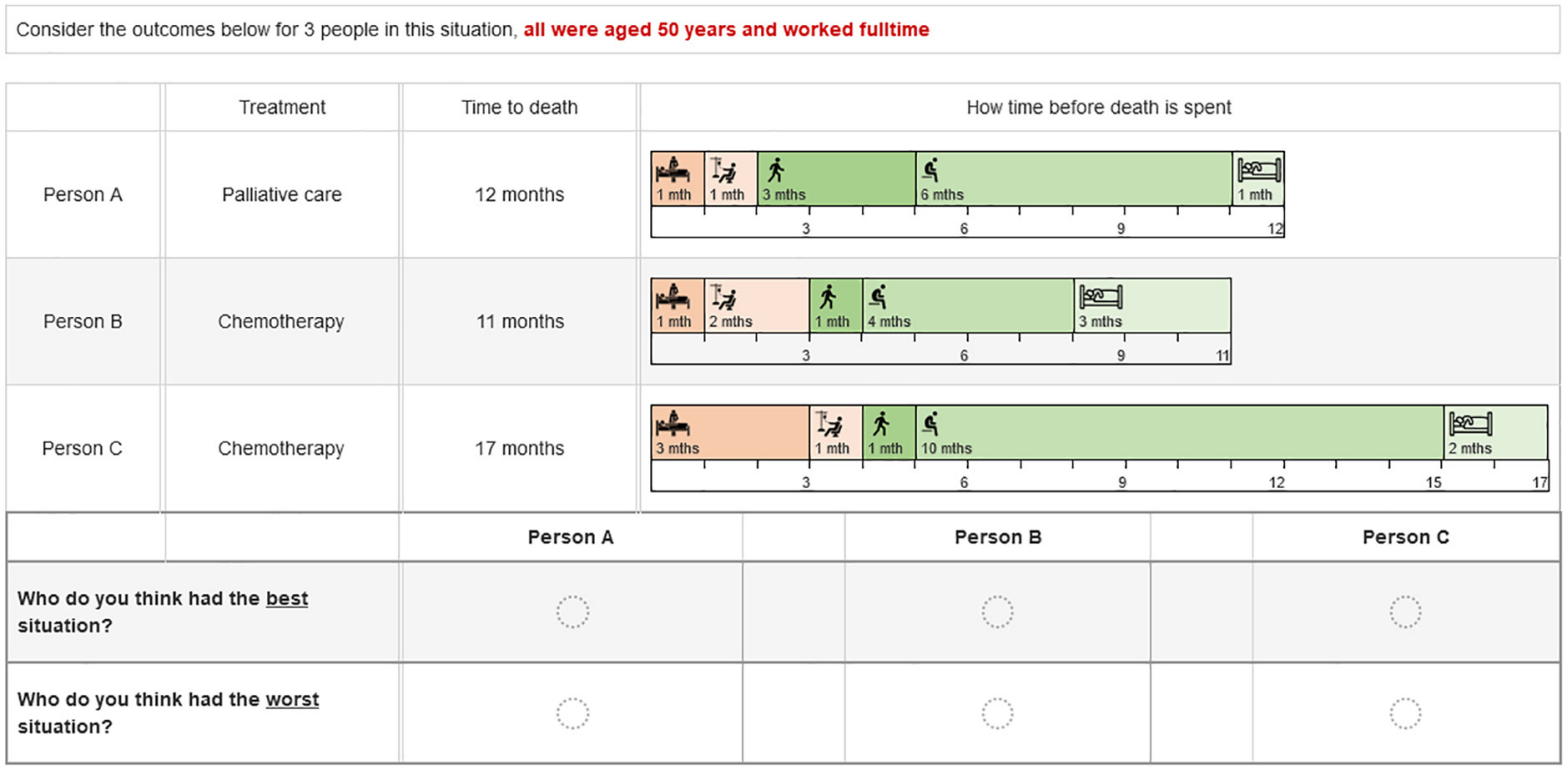
Example choice set.

The alternatives were presented as different people who chose either chemotherapy or palliative care (person A, person B, person C). Each choice set included a single fixed palliative care alternative and 2 chemotherapy alternatives with different attribute levels varying according to the design. The time periods were presented graphically with the choice sets including the treatment label (either palliative care or chemotherapy) and the overall survival, which was the sum of the attribute levels for that alternative. We could not include a separate independent attribute for overall survival time as it had to be consistent with the attribute durations. Respondents could hover over an attribute representation for a pop-up definition of that attribute. The attributes and levels are shown in Table 1, and an example of a choice set is shown in Figure 1. Respondents were randomized to see the palliative care alternative as person A or person C (position 1 or 3). As we thought that the age and life stage of the person with cancer might affect preferences, we included 3 patient vignettes describing people with cancer at age 35, 50, and 80 y. Each respondent saw 5 choice sets related to each vignette and were told to assume that all 3 people in the choice set were in the same situation at the time of choosing treatment. The order in which respondents saw the age vignettes was randomized. The overall situation description and the age vignettes are provided in the online material (page 1), along with an example full questionnaire.

**Table 1:** Attributes and Levels

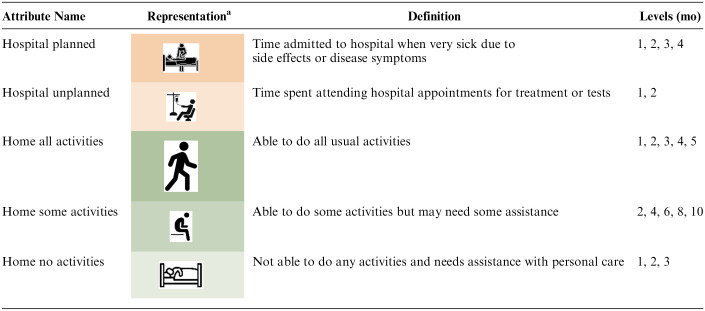

a The attribute definition was available in a pop-up.

### DCE Design

We used a generator-developed design^29^ in which the initial set of options came from the first 5 columns of the orthogonal array with 25 runs given by Kuhfeld^30^ and with the entries evaluated modulo the number of levels for the corresponding attribute. We insisted that in the set of 5 generators, there was 1 generator with a 0 entry for each attribute so that respondents could not make all decisions by focusing on 1 attribute only. The sixth column of the orthogonal array was used to form versions of size 5, with 1 choice set from each generator in each version. Versions of 15 choice sets were formed by using 3 different versions of size 5, 1 for each of the 3 age vignettes. The final design included 375 choice sets (25 versions of 15). In addition to randomizing the order in which respondents saw each age vignette, the order of the choice sets within each set of 5 was also randomized.

### Analysis

Stated preference studies are often used in policy analyses because individuals gain utility from the availability of certain social goods such as national parks, or, as in this case, the availability of the range of care options for the terminally ill that they deem most appropriate.^31^ Thus, the study will allow us to determine the relative importance of the attributes presented.

The multinomial logit (MNL) model and latent class analysis^32^ were used to analyze best choices. Only the analysis of the best choices is presented here as, like Huls et al.,^33^ we found that the inclusion of the second (worst) choices less consistent than the first (best) choices and did not pass the Swait–Louviere test^34^ for poolability (see online materials, page 2). The attribute levels were dummy coded, and the model included a constant for the palliative care alternative, along with interactions between the age vignette and the palliative care constant. Although respondents saw 3 alternatives (1 palliative care alternative and 2 chemotherapy alternatives), we included only 1 alternative specific constant for the palliative care alternative. As the attribute levels provided the only differences between the 2 chemotherapy alternatives, they were treated as generic alternatives.

In the MNL model, the utility derived by person *n* from choosing alternative *j* in scenario *s* is

(1) $$U_{\mathrm{njs}}=\beta x_{\mathrm{njs}}+\varepsilon_{\mathrm{njs}};n=1,\ldots ,N;\;j=1,\ldots ,J;s=1,\ldots ,S,$$

where $x_{\mathrm{njs}}$ is a vector of observed attributes of alternative *j*, β is a vector of utility weights (assumed homogeneous across individuals), and $\varepsilon_{\mathrm{njs}}$ is the idiosyncratic error assumed to be distributed i.i.d. extreme value. This provides the choice probabilities

(2) $$P_{\mathrm{njs}}=\mathrm{Prob}\left(y_{\mathrm{njs}}=1\right)=\mathrm{Prob}\left(U_{\mathrm{njs}}-U_{\mathrm{nls}}>0\right)\forall l\neq j$$

Under these assumptions, the probability of individual *n* choosing alternative *j* in scenario *s* is

(3) $$P_{\mathrm{njs}}=\exp(\beta x_{\mathrm{njs}})/\sum_{l=1}^{J}\exp(\beta x_{\mathrm{nls}})$$

Latent class analysis uses a discrete mixing distribution to account for preference heterogeneity. Each respondent is assumed to belong to one of *Q* latent classes where preferences differ between classes but are homogeneous within classes. Individual *n* belongs to class *q* with probability $\pi_{\mathrm{nq}}$ so that

$$\begin{array}{c} \beta =\beta_{q}\mathrm{with}\;\mathrm{probability}\;\pi_{\mathrm{nq}}\;\mathrm{for}\;q=1,\ldots ,Q,\\ \;\;\mathrm{where}\sum_{q}\pi_{\mathrm{nq}}=1\wedge \pi_{\mathrm{nq}}>0. \end{array}$$

The latent class analysis was used to identify groups with different preferences, in addition to a disengaged group with all coefficients constrained to zero. A subsequent MNL model was used to identify individual characteristics and attitudes associated with each preference class. Latent Gold version 5.1 (Statistical Innovations Inc.) was used for the latent class analysis, and Stata 18 (StataCorp LLC) was used for other analyses.

## Results

The 1,502 completed surveys represented 63% of 2,382 potential participants who started the survey, after screening out respondents using a phone or where the age gender quota was full. The sample demographic characteristics are shown in Table 2. The sample was similar to the Australian population in terms of age, gender, employment status, and state of residence. The sample overrepresented people with a university degree, those born in Australia, those living in major cities, and those identifying as Aboriginal or Torres Strait Islander. Most (70%) reported having had someone close to them die from a life-limiting illness. Respondents were asked about attitudes to care at the end of life; 70.6% agreed that terminally ill patients should be able to end their life with medical assistance, 63.3% said that they would prefer care that focused on comfort even if it meant not living as long, and 25.6% said that they would prefer care that focused on extending life even if this meant more pain and discomfort.

**Table 2:** Characteristics of Participants (Aged 18 y or Older) and Australian Population Aged 15 y or Older

Characteristic	Sample (*N* = 1,502), %	Australians,^a^ %
Female	51.0	51.0
Age, y		
18–24	10.7	11.0
25–34	18.3	18.4
35–44	17.1	17.7
45–54	16.4	16.1
55–64	14.5	14.9
65–74	12.4	12.0
75–84	7.3	7.1
≥85	3.3	2.7
Health good/very good/excellent	76.2	
Close person died from terminal illness	70.2	
Born in Australia	84.6	65.9
Aboriginal or Torres Strait Islander	13.8	3.0
Language at home English	96.0	
Married or defacto	55.8	
Working full- or part-time	67.8	61.5
Education, degree	50.0	28.5
Residential area		
Major cities	80.4	72.2
Regional	19.0	25.9
Remote	0.6	1.9
State/territory		
Australian Capital Territory	1.4	1.8
New South Wales	33.7	31.4
Northern Territory	0.1	0.9
Queensland	20.8	20.4
South Australia	7.6	7.1
Tasmania	2.8	2.2
Victoria	24.4	25.6
Western Australia	9.2	10.6

a Australian Bureau of Statistics (June 2022), Census of Population and Housing: Snapshot of Australia 2021, https://www.abs.gov.au/statistics/people/people-and-communities/snapshot-australia/2021.

Among the 1,502 respondents, 7.9% always chose the alternative with the longest overall survival as best regardless of the attribute levels, while 2.8% always chose the palliative care alternative and 10.1% always chose one of the chemotherapy alternatives (i.e., an active treatment). The latent class analysis included one class (class 1) in which all coefficients were constrained to zero, to identify inattentive respondents answering randomly. A 5-class latent class model was selected based on interpretability rather than model fitting, which continued to improve with additional classes, leading to small classes with similar preference patterns in some of the models. We decided that the best-fitting model with 9 classes would not be the most useful model in understanding the important variation among respondents. Further detail about model selection is available in the online materials (page 4).

The coefficients for the 5-class model are shown in Table 3. The mean of the respondent maximum class probabilities was 0.894 for this model (median 0.974), which was slightly better than the models with more classes. The inattentive class (class 1) had an average class membership probability of 29% in the 5-class model, and this was consistent across the different models, where the size of the zero-constrained class ranged from 27% to 29% across all models including 5 or more classes. There was also consistency across the individual respondents assigned to membership of this class, with 25% of the sample allocated to this class in all models (based on each respondent’s highest probability of class membership). The remaining 4 classes represented groups with different preferences where the class shares were 23.6% for class 2, 18.9% for class 3, 15.7% for class 4, and 12.7% for class 5.

**Table 3:** Latent Class Analysis: 5 Class Model with Class 1 Coefficients Constrained to Zero

		Class 1	Class 2		Class 3		Class 4		Class 5	
Attribute	Months	Coefficient	Coefficient	SE	Coefficient	SE	Coefficient	SE	Coefficient	SE
Hospital admitted (unplanned)	2	0	0.625***	0.117	−0.002	0.091	−0.700***	0.117	−0.100	0.082
	3	0	1.440***	0.133	−0.074	0.103	−0.820***	0.126	0.186*	0.086
	4	0	1.817***	0.138	−0.292**	0.101	−1.207***	0.148	0.116	0.087
Hospital attending (planned)	2	0	0.538***	0.068	−0.037	0.064	−0.114	0.090	−0.020	0.051
Home all activities	2	0	0.874***	0.140	0.450**	0.133	0.186	0.152	0.020	0.099
	3	0	1.818***	0.129	1.281***	0.129	0.128	0.157	0.112	0.095
	4	0	2.909***	0.167	2.102***	0.135	0.504**	0.159	0.267*	0.103
	5	0	3.992***	0.173	2.798***	0.150	0.791***	0.166	0.343**	0.105
Home some activities	4	0	1.592***	0.142	0.603***	0.133	−0.508***	0.132	0.146	0.099
	6	0	3.641***	0.175	1.346***	0.134	−0.709***	0.149	0.197	0.104
	8	0	5.225***	0.234	1.946***	0.145	−0.728***	0.160	0.365**	0.103
	10	0	7.052***	0.296	2.871***	0.171	−0.600**	0.169	0.567***	0.115
Home no activities	2	0	0.488***	0.087	−0.031	0.081	−0.717***	0.105	−0.055	0.068
	3	0	1.111***	0.103	−0.240*	0.090	−1.228***	0.136	−0.086	0.074
Constant PC alternative		0	−0.332	0.175	−0.045	0.152	1.257***	0.178	−2.226***	0.263
Age 50 vignette × PC interaction		0	−0.078	0.156	0.407***	0.108	0.102	0.118	0.819**	0.268
Age 80 vignette × PC interaction		0	−0.192	0.161	0.849***	0.110	0.102	0.116	0.455	0.274
Class constant		0	−0.208*	0.088	−0.428***	0.100	−0.615***	0.099	−0.824***	0.128
Class share		0.290	0.236		0.189		0.157		0.127	
Number of respondents	1,502									
Number of choices	22,530									
Log likelihood	−19,361									
AIC	38,867									
BIC	39,250									

AIC, Akaike’s information criterion; BIC, Bayesian information criterion; PC, palliative care; SE, standard error.

* *p* < 0.05; **p < 0.01; ***p < 0.001.

### Class Preferences

**Figure 2:**
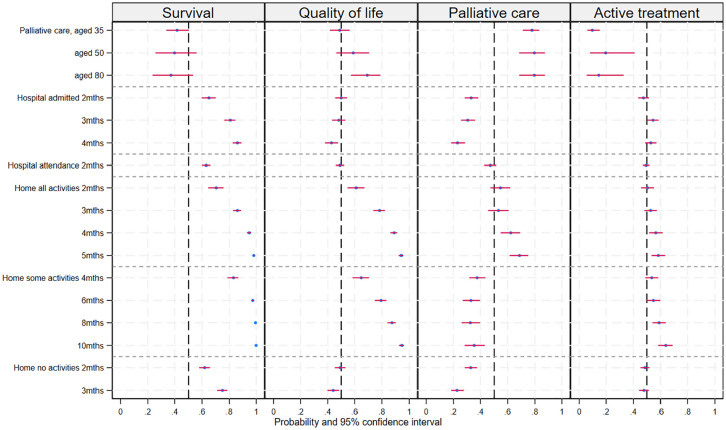
Predicted probability of choosing an alternative at each attribute level holding other attributes constant across alternatives. The dashed line at 0.5 represents indifference between alternatives with the attribute level or the base level (base 2 mo for home some activities and 1 mo for other attributes).

Class 2 appeared to represent a group with a strong preference for longer survival. For this class, all coefficients related to time in hospital or at home were statistically significant, positive, and increased with duration. The palliative care constant and the age vignette interactions were negative but not significantly different from zero for class 2 (see Table 3). The probability of choosing an alternative at each attribute level with other attributes constant is shown in Figure 2, which illustrates the increasing probability associated with longer durations irrespective of quality of life.

Class 3 appeared willing to trade quantity and quality of life, preferring longer periods with better functioning levels and a preference for palliative care for older patients. The coefficients for time spent at home being able to do all or some usual activities were positive, statistically significant, and increased with more time spent in these states (see Table 3). This group also preferred alternatives without longer periods in hospital or confined to bed at home. The preference for palliative care to treat older patients can be seen in Figure 2, where the probability of choosing the palliative care alternative increased with the age of the person described in the patient vignette. The probability of choosing an alternative at the maximum level of some and all activities was similar (see Figure 2), despite these attribute levels representing different time periods; the change from baseline represented an increase of 8 mo for some activities and 4 mo for all activities, suggesting that twice as much life extension was required to compensate for a reduction in capacity from all to only some usual activities.

The large, positive, and statistically significant palliative care constant with nonsignificant age vignette interactions (Table 3) suggests that class 4 represented a group with a strong preference for the palliative care alternative irrespective of the age of the patient. This group preferred longer time (4 or 5 mo) at home being able to do all activities but preferred alternatives that did not include time admitted to the hospital or time at home with any level of incapacity. This is illustrated in Figure 2, which shows a low probability of choosing an alternative for admitted hospital time and time at home able to do some or no activities.

Class 5 was the smallest class (share 12.7%) and had a large negative and statistically significant coefficient on the palliative care constant (Table 3), suggesting that this group preferred to use one of the active treatment alternatives (chemotherapy). There were few other statistically significant coefficients; these included the small positive coefficients on the longest periods spent at home being able to do all or some usual activities; however, these were very small relative to the preference to not use palliative care (see Table 3).

Among the 1,070 respondents whose highest class membership probability was not Class 1 (inattentive class), 33.5% were in class 2 (survival), 26.1% were in class 3 (quality of life), 22.5% were in class 4 (palliative care), and 17.9% were in class 5 (active treatment).

### Respondent Characteristics and Class Membership

Table 4 shows the MNL model to predict individual class membership, based on the highest membership probability. Relative to the survival preference group (class 2), both the inattentive group (class 1) and the active treatment group (class 5) were more likely to complete the survey within 5 min and were less likely to be aged 55 y or older. Class 1 was also less likely to have a university degree and more likely to report poor or fair health relative to class 2. Relative to class 2, the palliative care group (class 4) was more likely to have had someone close die from a life limiting illness.

**Table 4:** Respondent Characteristics and Attitudes Associated with Class Membership (Relative to Class 2^a^): Multinomial Logit Model

	Class 1: Inattentive		Class 3: Quality of Life		Class 4: Palliative Care		Class 5: Active Treatment	
	Coefficient	SE	Coefficient	SE	Coefficient	SE	Coefficient	SE
Completed survey <5 min	1.222***	0.285	−1.720*	0.756	0.091	0.470	0.915**	0.341
Aged 35–54 y	0.031	0.189	−0.007	0.221	0.242	0.250	−0.227	0.221
Aged 55 y or older	−0.801***	0.210	−0.367	0.231	0.021	0.251	−0.878**	0.253
Female	−0.161	0.151	−0.293	0.168	−0.016	0.179	−0.332	0.184
University degree	−0.573***	0.164	−0.380*	0.183	−0.490*	0.194	−0.380	0.201
Self-reported health fair/poor	0.761***	0.194	0.464*	0.212	0.125	0.224	−0.030	0.263
Someone close died from life-limiting illness	−0.044	0.164	0.358	0.188	0.444*	0.204	0.397	0.206
Attitudes to end-of-life care								
Agree with medical assistance to end own life	0.487**	0.176	0.278	0.197	0.358	0.220	−0.224	0.201
Patients owe loved ones to try treatments	−0.213	0.166	−0.335	0.182	−1.176***	0.205	−0.091	0.203
Would want sedation if pain too much	−0.665**	0.196	−0.108	0.234	0.048	0.259	−0.419	0.230
Would prefer care focused on extending life	−0.620***	0.173	−1.547***	0.234	−1.455***	0.276	−0.392	0.203
Constant	1.079***	0.265	0.375	0.310	−0.064	0.340	0.433	0.314
Observations	1,502							
Log likelihood	−2,167							

SE, standard error.

a Survival focus.

* *p* < 0.05; **p < 0.01; ***p < 0.001.

Attitudes to care at the end of life were also included in the class membership model. Relative to the survival group (class 2), both the quality-of-life group (class 3) and the palliative care group (class 4) were much less likely to say that they would prefer care focused on extending life if they had a terminal illness. The palliative care group were also less likely to believe that terminally ill patients owed it to their loved ones to try available treatments. The groups were similar for agreement with terminally ill patients being able to end their life with medical assistance, with the exception of the inattentive group which was more likely to support this. There were no statistically significant differences in attitudes to end-of-life care between the active treatment group (class 5) and the survival group (class 2).

## Discussion

This study assessed community preferences for care at the end of life and identified 4 groups with different preferences as well as an additional inattentive group who appeared to have responded to the DCE questions randomly. The 4 preference groups suggested differing values and preferred goals of care at the end of life. The largest group (survival group) placed great emphasis on extending overall survival regardless of how that time was spent. The second largest group (quality-of-life group) appeared willing to accept shorter survival time for the patient to spend less time in the hospital or completely incapacitated at home. This group was more likely to choose the palliative care alternative when the patient described was older. The third largest preference group (palliative care group) strongly supported the use of palliative care regardless of the age of the patients and preferred that the patient not spend time in hospital or at home with any functional limitations. The smallest group (active treatment group) was much less likely to choose the palliative care alternative, choosing one of the chemotherapy alternatives instead. The inattentive group was younger and more likely to complete the survey quickly relative to the other groups, with the exception of the active treatment group.

Responses to questions about opinions regarding care at the end of life (completed prior to the DCE questions) showed some consistency with the preference groups. Relative to the survival group, both the quality-of-life group and the palliative care group were less likely to prefer care that focused on extending life, especially if it increased pain and discomfort. The palliative care group was also less likely to agree that the terminally ill owed it to their loved ones to try treatments. The active treatment group expressed similar attitudes to the survival group, and all 4 groups did not differ significantly in terms of attitudes toward medical assistance to end life or wanting to be sedated if physical or emotional pain became too much.

Our preference groups are similar to those identified by Finkelstein et al.^35^ among cancer patients with the addition of a small group preferring not to use palliative care in our study. This similarity is despite important differences in our approach and the different cultural context. Our study used a larger sample from the general population and included how and where time was spent, rather than assuming a constant quality of life over the survival period. Although the age of the respondents in our study did not distinguish the groups, our quality-of-life group (class 3) preferred palliative care for older patients, which is consistent with findings from studies of patient decision making in cancer care in which older patients were more likely to focus on quality of life while younger patients were more willing to accept more aggressive treatments to extend survival.^36^

### Limitations

Our study has some limitations. We included overall survival as the sum of the periods in each condition, rather than as a separate attribute. This approach allowed us to separately identify the value of time spent in the different conditions. It meant that we were unable to estimate a preference for longer survival directly; instead, this was identified through an increasing preference for longer time periods in any state whether positive or negative. However, it is more realistic to offer increased survival in a particular health state and avoids inconsistencies between the attributes and overall survival. We recruited our general population sample through an online panel, which will often include a number of inattentive respondents motivated to complete many surveys quickly. We used latent class analysis to identify a group of disengaged respondents (in addition to preference groups among attentive respondents). This is similar to Jonker’s^37^“garbage class”; however, instead of the weighted mixed logit analysis, we used latent class analysis as we were expecting groups with contradictory end-of-life care preferences. The percentage of inattentive respondents was similar to that identified by other studies (for example, Jonker^37^). We did not ask respondents to choose for themselves prospectively; rather, they were asked their most preferred alternative from a set showing completed trajectories for hypothetical patients in the same initial situation who chose a different treatment resulting in different time trajectories in terms of survival, time in hospital, and time with disability. We do not know if these preferences were what they would want for themselves or someone close to them under the same circumstances or just what they think should be available for people in general under the circumstances. Regardless, they were considering specific circumstances varied according to an experiment rather than their own unmeasured individual situations, which would occur when posing a single question to a current patient or carer population (although our general population sample will include some current patients or carers). These preferences are therefore useful for understanding population preferences but are not predictive of what individuals would choose under conditions of uncertainty

### Implications for Health Policy

Patient-centered care is care that is responsive to patient preferences, that requires an understanding of those preferences, and whether there is variability across preferences and values across the population. The DCE approach is a useful research method for understanding how people, whether as individuals choosing for themselves or as citizens expressing social preferences, value the different aspects of treatment. It also allows the identification of groups whose preferences may vary. The study finds population subgroups with differing preparedness to accept functional limitations for life extension.

This heterogeneity is particularly important in the end-of-life context, in which survival time is limited, health states can change rapidly, and patient autonomy is highly valued. Previous DCE studies of treatment at the end of life have assumed the patient’s health state to be constant over a given survival period, when the reality involves different periods spent in different health states. This study adds to the existing literature by using end-of-life trajectories with periods of time spent in hospital and at home with different levels of incapacity depicted graphically.

Service planning involves setting performance goals and allocating resources. In terminal cancer care, it regularly involves tradeoffs between active treatment that may involve serious side effects with the goal of extending life and palliation with the goal of maximizing the quality of life. Typically, though, average preferences are used to set such goals, such as providing for deaths at home or the uptake of palliative care. As Nolte et al.^15^ pointed out, respect for patient preferences can be in conflict with standardized approaches to care. More work is required to investigate patient and societal values, to measure them robustly, and to reconcile these competing values. For now, our results provide guidance for setting performance goals. A simple approach such as prioritizing deaths at home or increasing palliative care does not respect the heterogeneity in preferences. Performance goals need to cover more than one approach to care at end of life.

Existing payment approaches, particularly fee for service, can inhibit the provision of care that is more patient centered. For example, hospital care is more likely to be covered by private insurance or public funding than community-based care is. That leaves community care to be covered by personal and family out-of-pocket spending. As well as encouraging less efficiency, this can also lead to more inequity in care provision. The focus on what service or what institution is paid limits the achievement of care that is integrated across several providers and is flexible in responding to patient and family preferences. This misalignment between many current payment approaches and the more patient-centered goals of end-of-life care has been described by others.^16,38^

There are alternative payment approaches that offer better incentives. For example, bundled care payments provide a fixed amount to cover a package of services. The package does not specify which services should be used but rather sets a payment envelope within which the services preferred by each patient can be supported. As each package would be adjusted for patient need, the available funds will be fairly allocated.

So, innovative funding approaches can better align payment with care objectives and provide high-value care, improving efficiency and equity. This is not to say that policy makers can easily and quickly implement innovative payments. There are several challenges in any implementation.^39^ We do see that the potential benefits of these would be high in helping improve palliative care. Policy makers can and should be exploring these funding approaches.

## Conclusion

Population preferences for active or palliative treatment with advanced cancer varied between different subgroups with contrasting preferences. We found a group focused on extending life and 2 groups willing to forgo longer life for less time in the hospital and less time with disability, one favoring palliative care and the other favoring palliative care in older patients. There was a remaining small group who preferred active treatment. For clinicians caring for people with a life-limiting illness, our results encourage the importance of understanding patients’ values and goals of care to ensure appropriate patient-centered care is delivered for people with life-limiting illness. For policy makers, this study demonstrates that there is substantial variability in end-of-life goals, and hence, it is important that policy objectives recognize this. Given the increasing support for patient-centered care, and given the substantial heterogeneity in preferences that we have found, a unidimensional goal such as increased referrals to palliative care would overlook patient-centeredness. This implies that any health service planning for end-of-life care, and the evaluation of such programs, should reflect multiple goals and are responsive to patients’ values. Further, innovative funding approaches such as bundled payments would provide a stronger patient-centered focus.
